# Assessment of the lung microbiota in dogs: influence of the type of breed, living conditions and canine idiopathic pulmonary fibrosis

**DOI:** 10.1186/s12866-020-01784-w

**Published:** 2020-04-10

**Authors:** Aline Fastrès, Elodie Roels, Emilie Vangrinsven, Bernard Taminiau, Hiba Jabri, Géraldine Bolen, Anne-Christine Merveille, Alexandru-Cosmin Tutunaru, Evelyne Moyse, Georges Daube, Cécile Clercx

**Affiliations:** 1 Department of Clinical Sciences, FARAH, Faculty of Veterinary Medicine, University of Liège, Liège, Belgium; 2 Department of Food Sciences – Microbiology, FARAH, Faculty of Veterinary Medicine, University of Liège, Liège, Belgium; 3 Department of Veterinary Management of Animal Resources/Biostatistics and Bioinformatics Applied to Veterinary Sciences, FARAH, Faculty of Veterinary Medicine, University of Liège, Liège, Belgium

**Keywords:** Lung, Microbiota, Dogs, CIPF, Breed, Environment

## Abstract

**Background:**

Literature about the lung microbiota (LM) in dogs is sparse. Influence of breed and living conditions on the LM in healthy dogs is currently unknown, as well as the influence of chronic respiratory diseases such as canine idiopathic pulmonary fibrosis (CIPF) in West highland white terriers (WHWTs). Aims of this study were (1) to assess the characteristics of the healthy LM according to breed and living conditions, and (2) to study LM changes associated with CIPF in WHWTs. Forty-five healthy dogs divided into 5 groups: domestic terriers (*n* = 10), domestic shepherds (*n* = 11), domestic brachycephalic dogs (*n* = 9), domestic WHWTs (*n* = 6) (H-WHWTs) and experimental beagles (n = 9) and 11 diseased WHWTs affected with CIPF (D-WHWTs) were included in the study to achieve those objectives.

**Results:**

In healthy domestic dogs, except in H-WHWTs, the presence of few discriminant genera in each type of breed was the only LM modification. LM of experimental dogs displayed a change in b-diversity and an increased richness compared with domestic dogs. Moreover, *Prevotella_7* and *Dubosiella* genera were more abundant and 19 genera were discriminant in experimental dogs. LM of both H-WHWTs and D-WHWTs revealed increased abundance of 6 genera (*Brochothrix*, *Curvibacter*, *Pseudarcicella*, Flavobacteriaceae genus*, Rhodoluna* and *Limnohabitans*) compared with other healthy domestic dogs*. Brochothrix* and *Pseudarcicella* were also discriminant in D-WHWTs compared with H-WHWTs and other healthy domestic dogs.

**Conclusions:**

In domestic conditions, except for H-WHWT, the breed appears to have minor influence on the LM. LM modifications were found in experimental compared with domestic living conditions. LM modifications in H-WHWTs and D-WHWTs compared with other healthy domestic dogs were similar and seemed to be linked to the breed. Whether this breed difference might be related with the high susceptibility of WHWTs for CIPF requires further studies.

## Background

The term “microbiota” refers to all the bacteria that are found in a particular region or habitat [1]. While the lung has been for long considered sterile, it is now well recognized that it hosts a diverse, low biomass bacterial population [2]. The lung microbiota (LM) in healthy experimental dogs is composed of a microbial population similar to the one in heathy humans, with major phyla including Firmicutes, Actinobacteria, Proteobacteria and Bacteroidetes [3–5]. In a pilot study from Roels et al. (2017), based on a limited number of dogs, LM differences were found between healthy experimental beagles (*n* = 6) and healthy client-owned West Highland white terriers (WHWTs) (*n* = 5) suggesting a possible association with the breed and/or the living conditions. In the same study, differences in the LM were also highlighted between healthy client-owned WHWTs and WHWTs affected with canine idiopathic pulmonary fibrosis (CIPF) (*n* = 7) suggesting an influence of the disease on the LM.

Whether the LM varies according to the type of breed in dogs is unknown. We hypothesize that inter-breed differences in genetic, morphological or physiological characteristics, or in breathing pattern could alter the LM which might be among the factors that favor lower airway diseases with breed predisposition.

The living conditions are suspected to play a role in the LM, although it has not yet been investigated in dogs. Indeed, the respiratory tract is in constant contact with the external environment which is known to be one of the factors that impacts the LM [2]. The influence of the living conditions on the LM has been specifically studied in horses and mice [6, 7]. In horses, it has been shown that lung communities are more similar between horses living in the same environment than living in different areas [6]. In mice, the LM clusters highly by cage, shipment and vendor suggesting a clear impact of the living conditions [7]. The living conditions could then represent an important source of variations of the LM and might also be among the factors able to predispose to lung diseases in dogs as it has been shown for asthma in human medicine for example [8–10].

CIPF is a poorly understood parenchymal lung disease mimicking notably idiopathic pulmonary fibrosis (IPF) in man. CIPF affects old dogs of the WHWTs breed, suggesting a breed predisposition for the disease [11, 12]. In man, numerous studies have been published supporting the hypothesis that the LM could be a trigger or a perpetuation factor in IPF [13–16]. In dogs, the alteration of the LM in CIPF dogs has not yet been investigated in a large number of animals.

The aims of this study were to assess [1] differences in the LM associated with the breed and the living conditions in healthy dogs; and [2] the LM alterations associated with CIPF in the WHWT breed. Therefore, the LM of healthy dogs from different breeds and living conditions was compared. Additionally, the LM of WHWTs affected with CIPF was compared with the LM of healthy WHWTs and of domestic dogs from other breeds.

## Results

### Influence of the breed on the LM

A total of 45 healthy adult dogs were included in the study and categorized into 5 groups according to the type of breed: terriers (T), shepherds (S), brachycephalic dogs (Br), WHWTs (H-WHWTs) and beagles (ExpB). Groups characteristics are reported in the Table 1. No age differences were found between groups (*P* = 0.052).

**Table 1:** Characteristics of the groups according to the type of breed

	**T**	**S**	**Br**	**H-WHWT**	**ExpB**
N	10	11	9	6	9
Sex (M/F)	6/4	3/8	5/4	4/2	4/5
Age, yr	7.01 (6.05–8.57)	6.96 (4.34–7.33)	3.61 (1.43–4.49)	8.68 (7.65–10.11)	4.82 (2.95–10.85)
Weight, kg	6.80 (5.55–9.18) ^a,b^	27.90 (23.35–31.15) ^a,c,d^	11.90 (9.50–13.30) ^c^	9.40 (8.65–9.70) ^d^	13.80 (12.70–16.20) ^b^
Breeds	7 Jack Russel, 3 Yorkshire	5 Belgian Malinois, 3 Australian shepherds and 1 white Swiss shepherd, 2 border collies	6 French and 1 English bulldogs, 1 pug, 1 Cavalier King Charles spaniel	WHWTs	Beagles

Data are expressed as median and interquartile range. Superscript letters reflect paired statistical difference (*P* < 0.002) according to Kruskal-Wallis and Dunn post-hoc tests. *M* Male, *F* Female, *T* Terriers group, *S* Shepherds group, *Br* Brachycephalic dogs group, *H-WHWT* Healthy West Highland white terriers group, *ExpB* Experimental beagles group

**Fig. 1:**
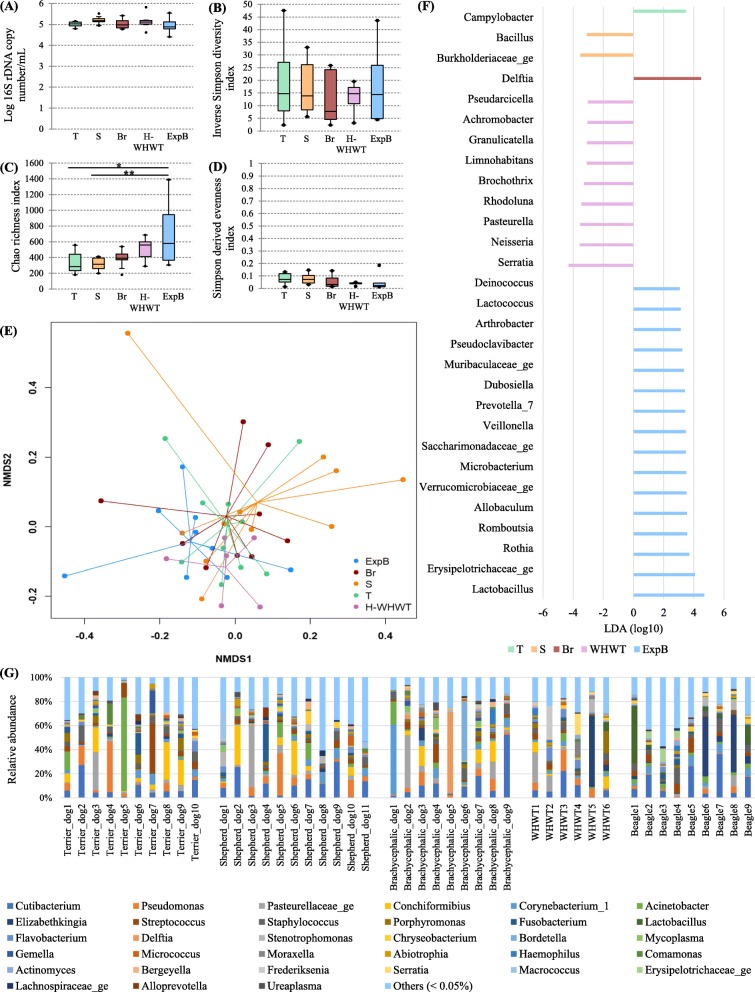
Influence of the type of breed on the lung microbiota. The influence of the type of breed on the lung microbiota was evaluated by comparison between 5 groups including domestic terrier dogs (T), domestic shepherd dogs (S), domestic brachycephalic dogs (Br), healthy domestic West Highland white terriers (H-WHWTs) and experimental beagles (ExpB). The parameters studied to assess the lung microbiota included; the bacterial load (**a**), the α-diversity (**b**), the richness (**c**), the evenness (**d**), the β-diversity (**e**) represented by a non-metric multidimensional scaling (NMDS) graph based on a Bray-Curtis matrix and the linear discriminant analysis (LDA) where only significant genera were represented (**f**). The distribution of the relative abundance of taxa at the genus level for each dog in each type of breed concerned only genera of more than 0.05% (**g**). *P = 0.002, **P = 0.001

The bacterial load in bronchoalveolar lavage fluids (BALFs) was not significantly different between the types of breeds (*P* = 0.22) (Fig. 1a).

Bacterial richness (Fig. 1c) was significantly higher in ExpB than in T and S groups, but there were no differences for the α-diversity and the evenness between groups (Fig. 1b and d; *P* = 0.87 and 0.14 respectively). The β-diversity (Fig. 1e) was different between the groups (*P* = 0.002) with differences between ExpB and T, S and Br (*P* = 0.04, 0.01 and 0.05 respectively).

Four major phyla were shared between the groups, including in descending order, Proteobacteria, Actinobacteria, Firmicutes and Bacteroidetes. At the genus level, inter-individual variability was observed between the dogs (Fig. 1g)) and a lot of genera were present in very small proportions (i.e., < 0.05%). The 10 most abundant genera in median across groups were *Cutibacterium*, *Staphylococcus*, *Streptococcus*, *Pseudomonas*, *Corynebacterium_1*, Pasteurellaceae genus, *Acinetobacter*, *Conchiformibius*, *Flavobacterium* and *Porphyromonas*. Taken together, all these genera represented 22.96% (9.59–56.64) of the global relative abundance across the groups. The linear discriminant analysis (LDA), used to determine the genera most likely to explain the differences between the groups [17], revealed a total of 29 genera, 1 in T, 2 in S, 1 in Br, 9 in H-WHWT and 16 in ExpB, which were discriminant between the groups (Fig. 1f). Significant differences in the relative abundance of the taxa at the genus level were only found between H-WHWTs and the other groups and are reported in the Table 2.

**Table 2:** Relative abundance of taxa at the genus level significantly different between the type of breed

**Genus**	**T**	**S**	**Br**	**H-WHWT**	**ExpB**	***P*-value**
*Brochothrix*	0% ^a^	0% ^b^	0% ^c^	0.28% (0.05–0.45) ^a,b,c^	0% (0–0.04)	^a,c^*P* < 0.05 ^b^*P* < 0.01
*Limnohabitans*	0% ^a^	0% ^b^	0% ^c^	0.24% (0.06–0.39) ^a,b,c,d^	0% ^d^	^a,b,c^*P* < 0.01 ^d^*P* < 0.05
*Rhodoluna*	0% ^a^	0% ^b^	0% ^c^	0.45% (0.09–0.95) ^a,b,c,d^	0% ^d^	^a^*P* < 0.001 ^b,c^*P* < 0.01 ^d^*P* < 0.05
*Curvibacter*	0% ^a^	0% ^b^	0% ^c^	0.06% (0.01–0.10) ^a,b,c,d^	0% ^d^	^a,b,c^*P* < 0.001 ^d^*P* < 0.01
*Pseudarcicella*	0% ^a^	0% ^b^	0% ^c^	0.20% (0.05–0.23) ^a,b,c,d^	0% ^d^	^a,b,c^*P* < 0.01 ^d^*P* < 0.05
Sporichthyaceae genus	0% ^a^	0% ^b^	0% ^c^	0.11% (0.01–0.18) ^a,b,c,d^	0% ^d^	^a,b,c^*P* < 0.001 ^d^*P* < 0.05

Results were expressed as median percentage of the relative abundance and interquartile range. Superscript letters reflect paired statistical difference by raw according to Kruskal-Wallis and Tukey post hoc tests. T group: terrier dogs; S group: shepherd dogs; Br group: brachycephalic dogs; H-WHWT: healthy West Highland white terrier dogs; ExpB group: experimental beagle dogs

### Influence of the living conditions on the LM

The LM has been then compared between dogs living in experimental versus domestic conditions. Domestic conditions were further divided into rural and urban conditions based on owner’s information. The same 45 dogs included in the first part of this study were used and re-categorized into the three living conditions (Table 3). No age difference was reported between groups.

**Table 3:** Characteristics of the groups according to the living condition

	**Rural**	**Urban**	**ExpB**
N	20	16	9
Sex (M/F)	8/12	8/8	4/5
Age, yr	7.01 (3.70–7.79)	6.58 (3.36–7.93)	4.82 (2.95–10.85)
Weight, kg	9.65 (7.10–13.45)	16.50 (9.70–29.08)	13.80 (12.70–16.20)
Breeds repartition	T: 9/10, S: 5/11, Br: 4/9, H-WHWT: 2/6	T: 1/10, S: 4/11, Br: 5/9, H-WHWT: 4/6	ExpB: 9/9

Data are expressed as median and interquartile range. *M* Male, *F* Female, *T* Terriers group, *S* Shepherds group, *Br* Brachycephalic dogs group, *H-WHWT* Healthy West Highland white terriers group, *ExpB* Experimental beagles group

**Fig. 2:**
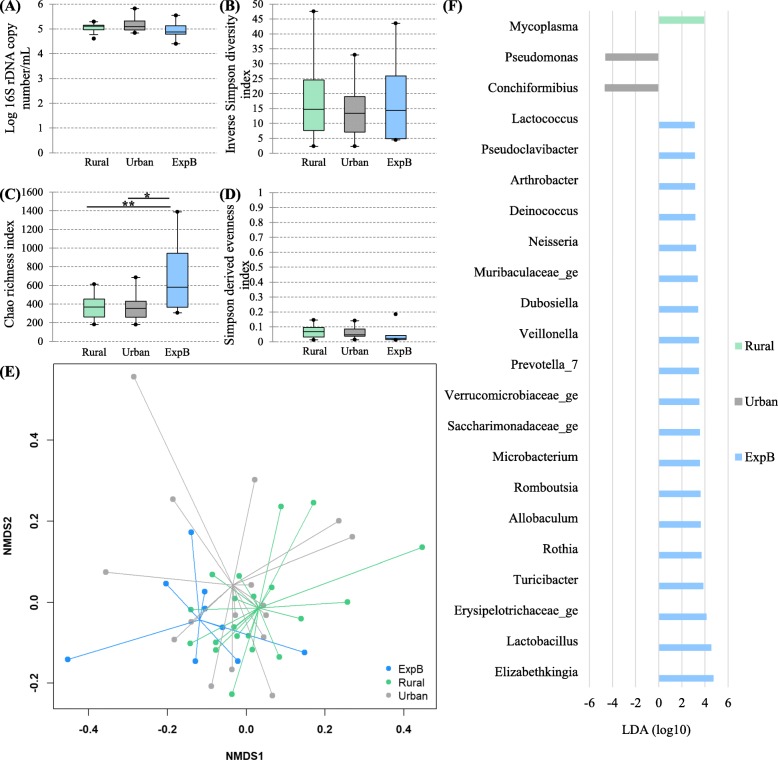
Influence of the living conditions on the lung microbiota. The influence of the living conditions on the lung microbiota was evaluated by comparison between domestic dogs living either in rural or urban condition and experimental beagle dogs (ExpB). The parameters studied to assess the lung microbiota included; the bacterial load (**a**), the α-diversity (**b**), the richness (**c**), the evenness (**d**), the β-diversity (**e**) represented by a non-metric multidimensional scaling (NMDS) graph based on a Bray-Curtis matrix and the linear discriminant analysis (LDA) where only significant genera were represented (**f**). *P = 0.017, **P = 0.015

The bacterial load (Fig. 2a) was not significantly different between the living conditions (*P* = 0.24).

There were no differences between living conditions for the α-diversity and the evenness (Fig. 2b and d; *P* = 0.93 and 0.24 respectively). The richness was higher in experimental compared with rural and urban conditions (Fig. 2c). The β-diversity (Fig. 2e) was different between the groups (*P* = 0.001) with significant differences between experimental and rural and between experimental and urban conditions (*P* = 0.003 and 0.039 respectively). No significant difference in the β-diversity was present between rural and urban conditions (*P* = 0.92). The LDA revealed a total of 22 genera, 1 in rural, 2 in urban and 19 in experimental conditions, which were discriminant between living conditions (Fig. 2f). Significant differences found between living conditions in the relative abundance of the taxa at the genus level are reported in the Table 4.

**Table 4:** Relative abundance of taxa significantly different between the living conditions

**Genera**	**Rural**	**Urban**	**ExpB**
*Prevotella_7*	0% (0–0.002) ^a^	0% ^b^	0.23% (0.18–0.33) ^a,b^
*Dubosiella*	0% ^a^	0% ^b^	0.04% (0–0.53) ^a,b^

Results were expressed as median percentage of the relative abundance and interquartile range. Superscript letters reflect paired statistical difference by raw (*P* < 0.05) according to Kruskal-Wallis and Tukey post hoc tests. *ExpB* Experimental beagle dogs

### The LM in CIPF WHWTs

To assess the LM in dogs affected with CIPF, we compared 11 WHWTs affected with CIPF (D-WHWTs) with age-matched healthy WHWTs and with all the other healthy domestic dogs (T, S and Br). The clinical characteristics of the groups are reported in the Table 5.

**Table 5:** Characteristics of the groups according to the disease status

	**Healthy domestic dogs other than WHWTs**	**H-WHWTs**	**D-WHWTs**
N	30	6	11
Sex (M/F)	14/16	4/2	4/7
Age, yr	6.25 (3.53–7.39) ^a^	8.68 (7.65–10.11)	11.52 (10.51–12.33) ^a^
Poids, kg	12.45 (7.68–23.68)	9.40 (8.65–9.70)	9.5 (9.25–10.45)

Data are expressed as median and interquartile range. Superscript letters reflect paired

statistical difference (*P* < 0.0001) according to Kruskal-Wallis and Dunn post hoc tests. *H-WHWTs* Healthy West Highland white terriers, *D-WHWTs* West Highland white terriers affected with canine idiopathic pulmonary fibrosis, *M* Male, *F* Female, *BALF* Bronchoalveolar lavage fluid

**Fig. 3:**
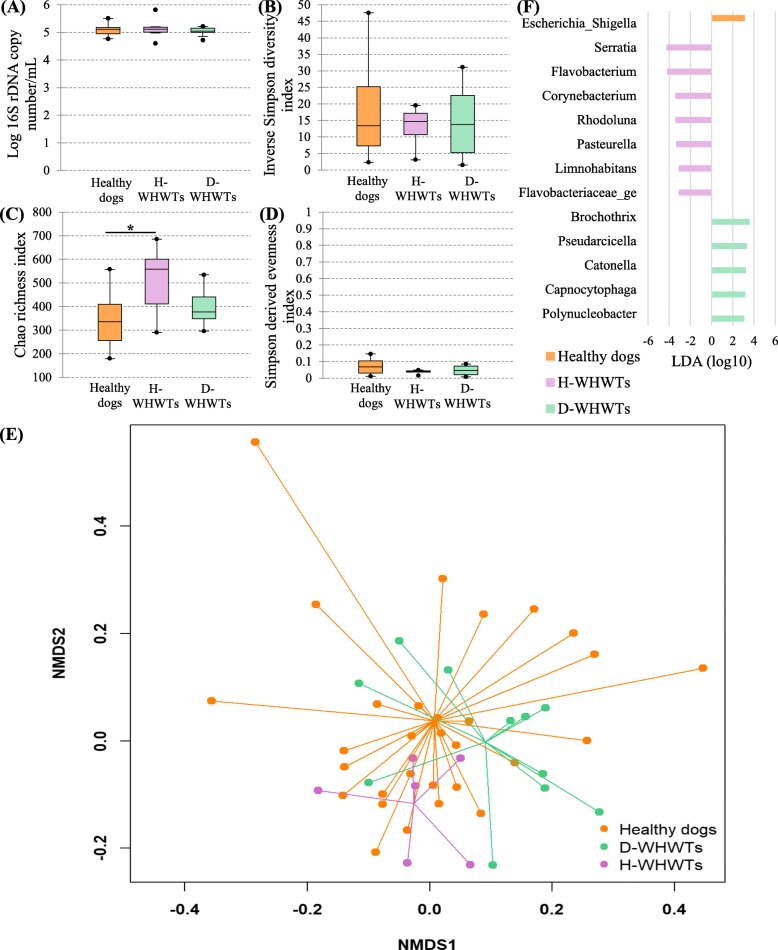
The lung microbiota in West Highland white terrier affected with canine idiopathic pulmonary fibrosis (CIPF). The influence of CIPF on the lung microbiota was evaluated by comparison between healthy domestic dogs from different breeds (healthy dogs), healthy West Highland white terriers (H-WHWTs) and WHWTs affected with CIPF (D-WHWTs). The parameters studied to assess the lung microbiota included; the bacterial load (**a**), the α-diversity (**b**), the richness (**c**), the evenness (**d**), the β-diversity (**e**) represented by a non-metric multidimensional scaling (NMDS) graph based on a Bray-Curtis matrix and the linear discriminant analysis (LDA) where only significant genera were represented (**f**). *P = 0.007

The bacterial load (Fig. 3a) was not different between the groups (*P* = 0.88).

There were no differences between groups for the α-diversity and the evenness (Fig. 3b and d; *P* = 0.86 and 0.13 respectively). The richness was significantly higher in H-WHWTs compared with healthy domestic dogs other than WHWTs (Fig. 3c). The β-diversity (Fig. 3e) was significantly different between the groups (*P* = 0.001) with significant differences only between D-WHWTs and healthy domestic dogs other than WHWTs (*P* = 0.003). No difference in the β-diversity was present between D-WHWTs and H-WHWTs (*P* = 0.18) and between H-WHWTs and healthy domestic dogs other than WHWTs (*P* = 0.13).

The LDA revealed a total of 13 genera, 1 in healthy domestic dogs other than WHWTs, 7 in H-WHWT and 5 in D-WHWT, which were discriminant between groups (Fig. 3f). Significant differences found between the groups in the relative abundance of the taxa at the genus level are reported in the Table 6.

**Table 6:** Relative abundance of taxa significantly different between the disease status

**Genera**	**Healthy domestic dogs other than WHWTs**	**H-WHWTs**	**D-WHWTs**	***P-*value**
*Limnohabitans*	0% ^a,c^	0.24% (0.06–0.39) ^a,b^	0.14% (0.06–0.34) ^b,c^	^a^*P* < 0.001 ^b^*P* < 0.01 ^c^*P* < 0.05
*Brochothrix*	0% ^a,b^	0.28% (0.05–0.45) ^a^	0.5% (0.29–0.92) ^b^	^a^*P* < 0.01 ^b^*P* < 0.001
*Curvibacter*	0% ^a,b^	0.06% (0.01–0.10) ^a^	0.07% (0–0.12) ^b^	^a^*P* < 0.05 ^b^*P* < 0.001
*Rhodoluna*	0% ^a,c^	0.45% (0.09–0.95) ^a,b^	0.27% (0.06–0.70) ^b,c^	^a^*P* < 0.001 ^b,c^*P* < 0.05
*Pseudarcicella*	0% ^a,b^	0.20% (0.05–0.23) ^a^	0.34% (0.04–0.58) ^b^	^a^*P* < 0.01 ^b^*P* < 0.001
Sporichthyaceae genus	0% ^a^	0.11% (0.01–0.18) ^a^	0.10% (0–0.22)	^a^*P* < 0.01
Candidatus Nomurabacteria genus	0% ^a^	0.16% (0.02–0.37) ^a,b^	0.06% (0.02–0.09) ^b^	^a,b^*P* < 0.001
*Serratia*	0% (0–0.02) ^a^	0.42% (0.18–1.60) ^a^	0.03% (0–0.13)	^a^*P* < 0.05
Flavobacteriaceae genus	0.01% (0–0.03) ^a,b^	0.23 (0.04–0.47) ^a^	0.26% (0.09–0.37) ^b^	^a^*P* < 0.001 ^b^*P* < 0.05

Results were expressed as median percentage of the relative abundance and interquartile range. Superscript letters reflect paired statistical difference by raw according to Kruskal-Wallis and Tukey post hoc tests. *H-WHWTs* Healthy West Highland white terriers, *D-WHWTs* West Highland white terriers affected with canine idiopathic pulmonary fibrosis

## Discussion

Results of the present study revealed that in healthy dogs, except for H-WHWTs and ExpB, the impact of the type of breed only seemed to concern the presence of few discriminant genera in each group. The LM in dogs living in experimental condition compared with the LM in dogs living in domestic condition was characterized by a change in the β-diversity with an increase of the bacterial richness. Moreover, *Prevotella_7* and *Dubosiella* genera were found in higher relative abundance and 19 genera were identified as discriminant in experimental compared with domestic living conditions. The LM in WHWTs either healthy or affected with CIPF was quite similar. In the LM of both healthy and diseased WHWTs, 6 genera were more abundant compared with the LM of healthy domestic dogs other than WHWTs and included *Brochothrix*, *Curvibacter*, *Pseudarcicella*, Flavobacteriaceae genus*, Rhodoluna* and *Limnohabitans. Brochothrix* and *Pseudarcicella* genera were also identified as discriminant genera in D-WHWTs.

In all samples from healthy dogs, four major phyla were detected, including in descending order, Proteobacteria, Actinobacteria, Firmicutes and Bacteroidetes. These four phyla are the same as those reported in previous studies in dogs [3–5] and also correspond to the major phyla found in the human LM, although not in the same order; in man Bacteroidetes and Firmicutes are the 2 most abundant [2]. *Cutibacterium* (previously named *Propionibacterium*), *Staphylococcus*, *Streptococcus*, *Pseudomonas*, *Corynebacterium_1*, Pasteurellaceae genus, *Acinetobacter*, *Conchiformibius*, *Flavobacterium* and *Porphyromonas* were the most abundant genera across all healthy dogs and represented together 22.96% of the LM at the genus level. Among these genera, Pasteurellaceae genus, *Pseudomonas*, *Acinetobacter*, *Cutibacterium*, *Streptococcus and Porphyromonas* were also found in high relative abundance in the study from Ericsson et al. (2016). In another study, the genera *Cutibacterium*, *Streptococcus*, *Staphylococcus*, *Pseudomonas*, *Corynebacterium_1* and *Acinetobacter* were part of the 10 most abundant genera composing the LM [5]. We therefore propose that the core genera of the LM in healthy dogs include at least the genera *Cutibacterium*, *Streptococcus, Acinetobacter* and *Pseudomonas*. *Cutibacterium* and *Streptococcus* genera contain Gram-positive anaerobic and aero-anaerobic bacteria respectively [18, 19]. In the study from Ericsson et al. (2016), *Cutibacterium* genus was only found in BALF samples, not in nasal, oropharyngeal and feces swabs. *Cutibacterium* genus is also commonly found in the healthy skin microbiota where it plays a role in skin homeostasis and in the protection against other harmful pathogen notably by pH reduction [18]. *Streptococcus* was reported as one of the main genera colonizing the oral and the lung microbiota in healthy but also in diseased patients [3, 19, 20]. Different strains of *Streptococcus* exist specialized in carbohydrate catabolism and environment acidification. Moreover, *Streptococcus* bacteria possess multiple high-affinity adhesins that mediate binding and biofilm formation preventing the growth of other potentially pathogen bacteria [19]. Bacteria in those 2 genera could exert same functions in the lung. *Pseudomonas* and *Acinetobacter* genera contain aerobic Gram-negative bacteria belonging to the Proteobacteria phylum. *Pseudomonas* genus represents a diverse group of bacteria characterized by a high ability to grow preventing the growth of other potentially pathogen bacteria and a capacity to produce and degrade a number of compounds including toxic materials for the host [21]. *Acinetobacter* bacteria could play an anti-inflammatory role in the LM. Indeed, a link was found between IL10 production, an anti-inflammatory cytokine, and *Acinetobacter* in asthma and atopic dermatitis [20]. However, *Streptococcus, Acinetobacter* and *Pseudomonas* genera are also involved in chronic infections particularly in ventilated patient and patient affected with chronic lung diseases. Knowing their capacity to form biofilms and resist to antimicrobial drugs [19–23]; indeed, in dogs under antimicrobial treatment It has been shown that *Pseudomonas* relative abundance increased [5]; it is important to take into account that those bacteria might be part of the core LM in dogs.

### Influence of the type of breed on the LM

The results of the LDA showed that few genera were identified as discriminant between groups of breeds, the number of discriminant taxa being more important in ExpB and H-WHWTs. No other modifications of the LM including in the relative abundance of the taxa, the bacterial load and the ecological parameters (diversity, richness and evenness) were identified between the groups except in ExpB and H-WHWTs. Those data indicate that in domestic healthy dogs other than WHWTs, the different types of breeds minimally alter the LM. That fact might be of interest when interpreting data about the microbiota in dogs from various breeds. The slight breed influence on the LM found in that study could be due to differences in the genetic or the morphology of the breeds as it has been shown in the gut where modification of the bacterial populations were described in relation with the dog’s size [24]. Since a clear link has been established between the gut microbiota and the LM [25], an influence, even slight, of the breed on the LM is not a surprising finding. Additional explanations for differences in LM encountered between breeds include differences in airways and lung anatomy and breathing strategy [26].

Of note, the fact that we included several breeds in the T, S and Br groups could have potentially masked certain breed-related differences. Moreover, it is also possible that the number of dogs included in each group was not sufficient to show more differences in the LM.

### Influence of the living conditions on the LM

The differences found between experimental and domestic living conditions overlap the influence of both the type of breed and the living conditions, as we were unable to recruit beagle dogs living in domestic conditions. In ExpB compared with T, S and Br, the β-diversity was different, the richness was higher and 16 discriminant genera were identified. When comparing dogs based on the living conditions (experimental vs domestic either rural or urban), similar changes were found; the β-diversity was different and the richness higher in experimental compared with domestic living conditions. More discriminant genera were identified in experimental conditions, including *Prevotella_7* and *Dubosiella* also found in higher relative abundance in experimental compared with domestic living conditions. Accordingly, we believe that the modifications of the LM in ExpB are most likely due to the living conditions rather than the breed. The importance of the close living conditions on the LM has already been reported in mice, where the LM highly clusters by cage, shipment and vendor [7] and in horses where lung communities are more similar between horses in the same environment than across environments [6]. In ExpB, the majority of the discriminant genera identified such as *Lactobacillus, Prevotella 7, Turicibacter* Verrucomicrobiaceae genus and Erysipelotrichaceae genus*,* are bacteria living in the intestinal tract and found in dog’s stools [27]. The presence of these bacteria might be linked to the living conditions of ExpB. Indeed, ExpB are housed by group on litter, in close contact with feces and they frequently exhibit a coprophagy behavior. Ingestion and sniffing of feces present in the direct environment of dogs most probably influence their LM as it results mainly from microaspiration, inhalation and direct dispersion along respiratory mucosa [2].

In domestic dogs, we did not show differences related to the fact that dogs were living mainly in either rural or urban area. However, it must be noted that this study was not designed to specifically assess the effect of such differences. Indeed, living areas in domestic dogs were arbitrary determined based on owner’s information. Additionally, if the global environment (rural or urban) of the domestic dogs was different, the housing conditions were quite similar when animals were inside house. By contrast, living conditions between experimental and domestic dogs strongly differed.

### The LM in CIPF WHWTs

*Brochothrix*, *Curvibacter*, *Pseudarcicella* and Flavobacteriaceae genus were found in higher relative abundance in healthy and diseased WHWTs compared with other healthy domestic dogs. Moreover, those genera were more abundant in D-WHWTs compared with H-WHWTs although not significantly. *Brochothrix* and *Pseudarcicella* genera were also discriminant for D-WHWTs. *Rhodoluna* and *Limnohabitans* genera were also found in higher relative abundance in WHWTs compared with domestic dogs from other breeds, but were higher in H-WHWTs than in D-WHWTs. These bacteria preferentially contaminate food (*Brochothrix* [28]) and water (*Curvibacter* [29], *Rhodoluna* [30], *Limnohabitans* [31], and Flavobacteriaceae_genus [32]), and can be ingested by dogs. Their presence in the LM of WHWTs could be associated with the higher abundance of gastroesophageal reflux reported in that breed compared with other breeds [33]. The higher rate of gastroesophageal reflux in WHWTs has been hypothesized to play a role in the onset and/or in the progression of CIPF [33]. In man, gastroesophageal reflux is associated with IPF although the role of these exposures in the pathogenesis of IPF is still only hypothetical [34].

A pathogenic role of *Rhodoluna*, *Limnohabitans Brochothrix*, *Curvibacter*, *Pseudarcicella* and Flavobacteriaceae genus bacteria in the lung has only been reported for bacteria of the Flavobacteriacea genus [32]. Indeed, some genera of the Flavobacteriaceae family, are known as opportunistic pathogen notably in human and animal such as *Elizabethkingia spp.* for example [32]. Bacteria of the Flavobacteriaceae family present different mechanisms of pathogenicity including cellular adhesion, gliding motility, proteolytic activity and resistance to immune system and antimicrobial drug [32]. Bacteria of the *Curvibacter* genus can expressed genes involved in carbon metabolism and fatty acid degradation, which is useful for the colonization of the mucus layer present in the lung [35]. They also had a flagellar structure able to promote their motility, their adherence and their penetration of mucosal barriers but also able to act as an activator of the immune system via Toll-like receptor signaling [29, 35]. Therefore, the presence in the LM of WHWTs of these 2 genera could activate the immune system, alter the airway epithelium and induce or perpetuate the airway inflammation in CIPF. Little is known about the metabolism and the potential pathogenic role of bacteria of the *Brochothrix*, *Pseudarcicella*, *Rhodoluna* and *Limnohabitans* genera [28, 30, 31, 36–38].

Data of the present study showed that both healthy and CIPF WHWTs had a distinct microbiota compared with other healthy domestic dogs. The majority of the genera found in higher relative abundance in H-WHWTs compared with other healthy domestic dogs were also found in higher relative abundance in D-WHWTs. This is in favor of the hypothesis that the LM modifications in WHWTs are most likely related to the breed. Accordingly, the LM might be among the factors able to predispose to CIPF. That hypothesis is supported by the fact that in mice with bleomycin-induced lung fibrosis, the dysbiosis precedes the peak of lung injury and persists in the fibrotic lung [39].

In human medicine, the LM in IPF patients was clearly modified compared with the LM of healthy patients on the contrary of what we observed in D-WHWTs compared to H-WHWTs. In IPF, the diversity decreased and the bacterial load increased in association with the disease progression, the presence of certain genera, not found in CIPF dogs, has also been associated with IPF such as *Haemophilus*, *Streptococcus*, *Neisseiria, Staphylococcus* and *Veillonella* [2, 39–44]. Of course, human and dogs are 2 different animal species with their own LM specificities. Moreover, the lack of common LM disturbances between IPF and CIPF may be related to the fact that the diseases, although presenting similarities, are different as suggested by well stablished differences in thoracic high resolution computed tomography (HRCT) [11, 45] and histopathological [46] characteristics.

## Conclusions

In domestic conditions, at the exception of the WHWT, differences between the types of breeds appear to have minor influence on the LM and should only minimally impact results of clinical studies comparing dogs of different types of breeds.

Differences in living conditions (experimental vs domestic dogs) were associated with modifications of the LM including a shift in the β-diversity, higher richness and identification of a large number of discriminant genera. Such differences need to be taken into account when results on the LM in experimental conditions are extrapolated to domestic dogs.

The LM found in WHWTs was distinct from the LM of other healthy domestic dogs. Therefore, LM changes in WHWTs seem to be associated with the breed. No clear modifications of the LM was found in CIPF compared with healthy WHWTs. Accordingly, the differences in the LM found in WHWTs might be related to the high susceptibility of that breed for CIPF and don’t seem to be induced by the disease. Further long-term follow-up studies before and during the course of CIPF and studies assessing the role of the bacteria specifically found in D-WHWTs including *Brochothrix*, *Curvibacter*, *Pseudarcicella* and Flavobacteriaceae genus are needed to assess whether the LM play a role as a trigger of CIPF and as a contributor for the perpetuation of the disease.

## Methods

### Study population

The study was performed at the veterinary clinic of companion animals of the University of Liège (CVU, Liège, Belgium). Adult dogs were prospectively included and classified into different groups based on their types of breeds, living conditions and disease status. The different breeds recruited included experimental beagles (ExpB), domestic shepherds (S), domestic terriers (T), domestic brachycephalic dogs (Br) and domestic healthy WHWTs (H-WHWTs). H-WHWTs were selected in view of their high predisposition for CIPF; ExpB dogs were chosen for their experimental living conditions; T were recruited because they belong to the same kind of breed and are morphologically similar to WHWTs but are not predisposed to CIPF; S were chosen because they are larger, athletic dogs with a thin and deep thorax; Br were selected based on their facial morphology causing a partial upper airways obstruction and a different pattern of breathing [26, 47, 48]. The living conditions were classified into experimental and domestic conditions. Besides, domestic conditions were further subdivided into rural or urban. For investigation of LM alterations associated with the disease, the group including WHWTs affected with CIPF (D-WHWTs) was compared to the group of H-WHWTs, and to a group including all the other healthy domestic dogs (T, S and Br groups combined).

Samples from healthy domestic dogs and dogs affected with CIPF were collected between May 2017 and April 2019. All dogs were privately owned (i.e. domestic dogs) and were housed in house or apartment, with or without outdoor access, and fed with various diets. For healthy dogs, owners were asked whether they lived in urban (inside a city of more than 2000 inhabitants) or rural area (in the countryside or in villages of less than 2000 inhabitants). Samples from ExpB were collected in December 2017. The beagles were experimental dogs housed in the experimental kennel of the University of Liège, by pair on woodchip litter, with a large concrete outdoor access, along a heavily used highway road 3 to 6 h per day and fed with a standardized commercial food.

At inclusion, the status of the dogs was confirmed based on clinical signs, physical examination, haematology and plasma biochemistry (Idexx, Hoofddorp, The Nertherlands), gross appearance of the respiratory tract during bronchoscopy and analysis of the BALF. Moreover, in WHWTs, CIPF was diagnosed or excluded according to a previously described approach [11] based on history, presence or absence of marked crackles on lung auscultation, and compatible findings or absence of abnormalities on thoracic HRCT. Dogs under antimicrobial drug or corticoids were not included in the study.

### Sample collection

BALFs were obtained in each dog under anesthesia as previously described [5]. Briefly, dogs were anesthetized by a veterinary anesthetist with a premedication with butorphanol 0.2 mg/kg intravenously (Butomidor® 10 mg/mL, Richter Pharma AG, Wels, Austria). Anesthesia was then induced and maintained with intravenous propofol (Diprivan® 1%, Asen Pharma Trading Limited, Dublin, Ireland) infusion on demand; animals were not intubated except WHWT dogs which were intubated with a sterile endotracheal tube and maintained under anesthesia with isoflurane during the HRCT before being extubated for the bronchoalveolar lavage (BAL) procedure. Before each use, the flexible pediatric endoscope (FUJINON© Paediatric Video-Bronchoscope EB-530S) used for the bronchoscopy was cleaned and disinfected. Then, 3 to 4 mL/kg of sterile saline solution were injected into the bronchoscope channel wedged in the respiratory tract of dogs and directly reabsorbed in a sterile recipient. BALFs were stored just after the collection without any processing at − 80 °C until DNA extraction. Dogs were awakened after the BAL procedure and returned to their owners for domestic dogs or brought back to the kennel for experimental dogs.

### DNA extraction

Analysis of the LM was performed as previously described [5]. Briefly, total DNA was extracted from BALFs according to manufacturer’s instructions with the DNeasy Blood and Tissue kit (QIAGEN Benelux BV, Antwerp, Belgium), using the pre-treatment for Gram-positive bacteria protocol. This protocol was preceded by a beat beating step with glass beads > 106 μm and glass beads soda-lime (Sigma-Aldrich, Overijse, Belgium, Cat. G4649 and Z265926). DNA was eluted into DNase/RNase-free water for a final volume of 30 μL and the concentration and purity were evaluated using a ND-1000 spectrophotometer (NanoDrop ND-1000, Isogen, De Meern, The Netherlands). All DNA extractions were done at the same time with the same kit and procedure.

### 16S quantitative PCR

The bacterial load was assessed in all samples by duplicate quantitative polymerase chain reactions (qPCRs) targeting the V2-V3 region of the 16S rDNA with the following primers, forward (5′-ACTCCTACGGGAGGCAGCAG-3′) and reverse (5′-ATTACCGCGGCTGCTGG-3′) [49] as previously described [5]. Results obtained were then expressed in base 10 logarithm of the total 16S rDNA copy numbers per mL.

### 16S rDNA library preparation, sequencing and informatics

For bacterial identification, polymerase chain reactions (PCRs) targeting the V1-V3 hypervariable regions of the 16S rDNA were performed for all samples at the same time with the following primers: forward (5′-GAGAGTTTGATYMTGGCTCAG-3′) and reverse (5′-ACCGCGGCTGCTGGCAC-3′) and Illumina overhand adapters as already described [5, 50]. Amplicons obtained were purified, quantified and submitted to a second PCR round for indexing. A final quantification of all samples was performed by qPCR before normalization and pooling of the amplicons to form libraries. Libraries obtained were then sequenced on a MiSeq Illumina sequencer (Illumina, San Diego, CA, USA) using V3 reagents. After sequencing, 8,930,807 reads with a median length of 508 nucleotides were obtained.

After a first cleaning step (length and sequences quality), 6,991,349 reads were screened for chimera using Vsearch algorithm [51]. Six million six hundred ninety-nine thousand five hundred seventy-two reads were retained for alignment and clustering using MOTHUR v1.40 [52]. Taxonomical assignation with an operational taxonomic unit (OTU) clustering distance of 0.03 were based on the SILVA database v1.32. A final subsampling was performed with a median reads per sample of 10,000.

### Identification of procedural contaminants and validation of the sequencing

Procedural control specimens (PCSs) were obtained in order to evaluate the general contamination during sampling just before each BAL by injection and direct reabsorption into the bronchoscope channel of 10 mL of sterile saline solution (NaCl 0.9%). PCSs were then stored at − 80 °C until further analysis. PCS were then treated exactly the same way as the BALF samples. Briefly, DNA was extracted with the same technique, same extraction kit and at the same time as samples. Bacterial load in PCSs was obtained by duplicate qPCRs performed in the same run as samples, by the same technique. The bacterial load was about 100 times lower in the PCS than in the corresponding BALF samples (*P* < 0.0001). PCR targeting the V1-V3 region of the 16S rDNA were performed as for the samples. The PCS amplification products were < 1 ng/μL and were then not sequenced.

A positive control using 20 defined bacterial species DNA and a negative control from the PCR step were included into the run to validate the sequencing.

### Statistical analysis

The influence of the type of breed was assessed by comparing the 5 groups of healthy dogs. The influence of the living conditions was evaluated by comparing experimental, rural and urban conditions. Finally, the influence of CIPF on the LM was evaluated by comparing all healthy domestic dogs other than WHWTs, H-WHWTs and D-WHWTs.

Normality was first checked using the Shapiro-Wilk test for each analysis. Characteristics of the groups were compared using Kruskal-Wallis test and post-hoc Dunn tests with Bonferroni correction using XLStat (Addinsoft, Paris, France).

Statistical differences in the relative abundance of taxa were assessed with Kruskal-Wallis test using Benjamini Hotchberg procedure and a false discovery rate of 10% for multiple comparisons, followed by Tukey-Kramer post hoc tests, using STAMP software [53]. The LDA was performed to detect discriminant bacteria between groups at the genus level with MOTHUR v1.40. For the LDA interpretation, differences were considered as significant for a *P* < 0.05 and a LDA score > 3.0 [17].

Metrics of richness (Chao1 index), evenness (Simpson index-based measure) and α-diversity (inverse Simpson’s index) were assessed with MOTHUR v1.40 at the OTUs level and compared between groups using Kruskal-Wallis test and Dunn post-hoc tests with Bonferroni correction using XLStat. The same test was used to compared the bacterial load between groups. Non-metric multidimensional scaling (NMDS) figures were performed based on a Bray-Curtis dissimilarity matrix at the OTUs level to represent the global bacterial composition (β-diversity) between groups (R vegan package). The β-diversity between types of breeds, living conditions and disease status was calculated using R (vegan package) by a permutational multivariate analysis of variance (PERMANOVA) followed by pairwise tests with Bonferroni correction.

Results are expressed in median and interquartile range. A *P*-value < 0.05 was considered as statistically significant.

## Data Availability

All sample raw reads associated with this study have been deposited at the National Center for Biotechnology Information (NCBI) under the accession number PRJNA594816.

## Abbreviations

LM: Lung microbiota
WHWT: West Highland white terrier
CIPF: Canine idiopathic pulmonary fibrosis
IPF: Idiopathic pulmonary fibrosis
S: Shepherd dogs
T: Terrier dogs
Br: Brachycephalic dogs
H-WHWT: Healthy West Highland white terrier
ExpB: Experimental beagles
BALF: Bronchoalveolar lavage fluid
LDA: Linear discriminant analysis
D-WHWT: West Highland white terrier affected with canine idiopathic pulmonary fibrosis
HRCT: High-resolution computed tomography
BAL: Bronchoalveolar lavage
qPCR: Quantitative polymerase chain reaction
PCR: Polymerase chain reaction
OTU: Operational taxonomic unit
PCS: Procedural control specimen
NMDS: Non-metric multidimensional scaling
PERMANOVA: Permutational multivariate ANOVA
